# Core principles of Malakit intervention for transferability in other contexts

**DOI:** 10.1186/s12936-024-05002-0

**Published:** 2024-06-13

**Authors:** Maylis Douine, Yann Lambert, Muriel Suzanne Galindo, Irene Jimeno Maroto, Teddy Bardon, Lorraine Plessis, Louise Mutricy, Jane Bordallo-Miller, Mathieu Nacher, Antoine Adenis, Hedley Cairo, Hélène Hiwat, Stephen Vreden, Carlotta Carboni, Alice Sanna, Martha Suarez-Mutis

**Affiliations:** 1 Centre d’Investigation Clinique Antilles-Guyane, INSERM CIC 1424, Cayenne Hospital, Cayenne, French Guiana; 2 DPAC-Fronteira, Oiapoque, Brazil; 3 National Malaria Elimination Programme, Ministry of Health of Suriname, Paramaribo, Suriname; 4 Foundation for the Advancement of Scientific Research, Paramaribo, Suriname; 5 Laboratory of Parasitic Diseases, Institute Oswaldo Cruz/Fiocruz, Rio de Janeiro, Brazil

**Keywords:** Transferability, Complex intervention, Sustainability, Implementation science

## Abstract

To eliminate malaria, all populations must be included. For those who are not reached by the health care system, specific interventions must be tailor-made. An innovative Malakit strategy, based on the distribution of self-diagnosis and self-treatment kits, has been evaluated in the Suriname-French Guiana- Amapá (Brazil) region. The results showed effectiveness and good acceptability. The Malakit intervention is complex and has many components. Its transferability requires adaptation to other populations and regions, while retaining the main features of the intervention. This article provides the keys to adapting, implementing and evaluating it in other contexts facing residual malaria in hard-to-reach and/or mobile populations. The process of transferring this intervention includes: diagnosis of the situation (malaria epidemiology, characteristics of the population affected) to define the relevance of the strategy; determination of the stakeholders and the framework of the intervention (research project or public health intervention); adaptation modalities (adaptation of the kit, training, distribution strategy); the role of community health workers and their need for training and supervision. Finally, evaluation needs are specified in relation to prospects for geographical or temporal extension. Malaria elimination is likely to increasingly involve marginalized people due to climate change and displacement of populations. Evaluation of the transferability and effectiveness of the Malakit strategy in new contexts will be essential to increase and refine the evidence of its value, and to decide whether it could be an additional tool in the arsenal recommended in future WHO guidelines.

**Supplementary Information:**

The online version contains supplementary material available at 10.1186/s12936-024-05002-0.

## Background

**Fig. 1:**
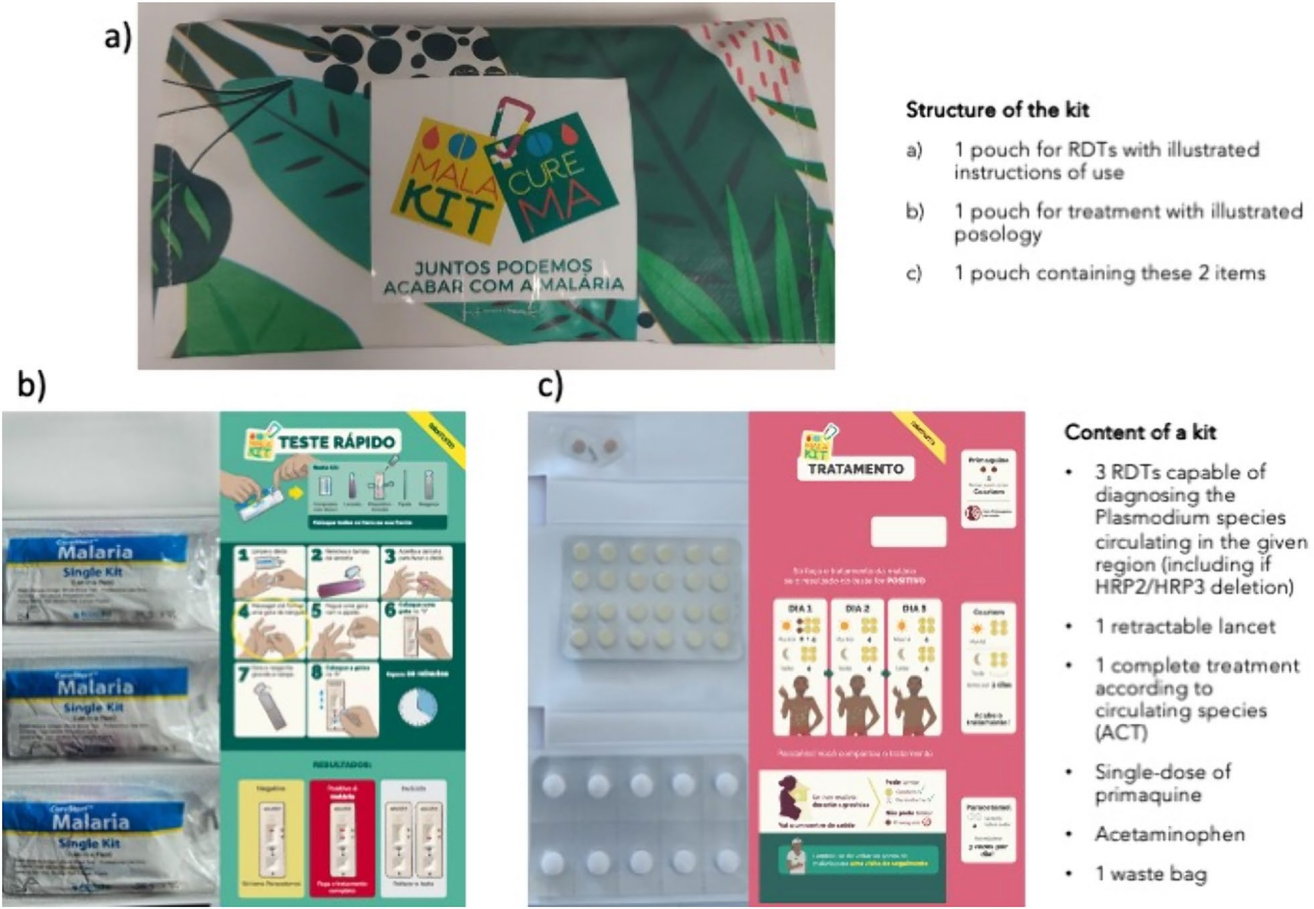
Presentation of a Malakit and its content

In the Guiana Shield, people working in artisanal and small-scale gold mining (ASGM) are heavily affected by malaria and represent a key transmission hub, hampering efforts to eliminate malaria in the region [1, 2]. In Suriname, specific programmes based on Community Health Workers (CHWs) have proved successful, but could not be implemented in French Guiana, a French overseas territory, for regulatory, security, and geographic reasons. An innovative strategy called Malakit overcame these hurdles and implemented the distribution of self-diagnosis and self-treatment kits by trained CHWs (Fig. 1) [3].

The objective of the intervention was to provide the targeted population with the equipment and resources needed to handle an episode of malaria symptoms by themselves when they are in a very remote location. The kits were distributed by CHWs specifically trained for the project. These CHWs came from the same community, mostly Brazilians from Northern and Northeastern states, spoke the same language and were based at strategic crossing points where these migrants cross the border to work in French Guiana [4, 5]. The 2-year research project (2018–2020) used an effectiveness evaluation by quasi-experimental design. The intervention was associated with an improvement in number of important outcomes: there was a significant increase of reliance on rapid malaria diagnosis before taking certified artemisinin-based combination therapy (ACT) (from 54.2% to 68.1%; OR = 1.8 (95% CI [1.1–3.0]); the intervention was followed by a significant decrease of malaria prevalence (on the border with Suriname) and of malaria cases exported from French Guiana to Suriname and Brazil, with an acceleration of the decline in malaria incidence in the region by 42.9% between 2018 and 2020 [6–8]. No safety or ethical concerns (such as significant resale on the black market) were reported. The strategy has been very well accepted by the ASGM community, with 30% of the population (estimated at 10,000 people) having received at least one kit during the two years of the intervention [6, 9, 10]. This represents an acceptable proportion in this context of very high mobility and with only five fixed distribution sites. The different methods of evaluation produced similar results, so that the convergence of evidence allows us to conclude that the strategy was effective.

Residual malaria in hard-to-reach populations is a global problem that challenges public health authorities, and new strategies are needed. Given the good acceptability, safety and effectiveness of the Malakit strategy, the question of its transferability to other contexts is worth raising. The transferability has been defined as the extent to which the measured effectiveness of an applicable intervention could be achieved in another setting [11]. It will then be useful to evaluate this strategy in other settings. Indeed, to achieve the World Health Organization (WHO) objectives of reducing malaria morbi-mortality by 90% by 2030 "no one must be left behind" notably cross-border or hard-to-reach populations—the exact purpose of the Malakit approach.

The lessons from the difficulties and successes encountered in the design, implementation and evaluation of the Malakit approach have been drawn and detailed in several articles (Table 1). All the documentation on this experience has been made accessible so that it can be used by other stakeholders.

**Table 1:** Publications presenting the different aspects of the Malakit interventional project with description of their main content

	Article	Content	References
1	Douine M, Sanna A, Galindo M, Musset L, Pommier de Santi V, Marchesini P, et al. Malakit: an innovative pilot project to self-diagnose and self-treat malaria among illegal gold miners in the Guiana Shield. Malar J. 2018;17:158	Description of the need for a new strategy to adress malaria in the ASGM in French Guiana	[3]
		Stakeholders and coordination	
		Concept of the Malakit strategy: content of the kit, training, distribution sites and design for evaluation	
2	Galindo MS, Lambert Y, Mutricy L, Garancher L, Bordalo Miller J, Gomes JH, et al. Setting-up a cross-border action-research project to control malaria in remote areas of the Amazon: describing the birth and milestones of a complex international project (Malakit). Malar J. 2021;20:216	Description of the Malakit project phases: feasibility, development, implementation	[4]
		Description in details of the choice of distribution sites, recruitment and training of CHWs, content of the IEC tools created, steps of first visit and follow-up visit	
		Details on coordination and cooperation organization, regulatory and administrative aspects of the study, logistics circuit, and participant safety	
3	Lambert Y, Galindo M, Suárez-Mutis M, Mutricy L, Sanna A, Garancher L, et al. Tailoring Mobile Data Collection for Intervention Research in a Challenging Context: Development and Implementation in the Malakit Study. JMIR Form Res. 2022;6:e29856	Description of the design, development and implementation of the Malakit information system for mobile data collection and monitoring	[19]
		Adaptation to field constraints (environment, internet bandwidth, electrical supply…)	
		Presentation of a homemade app fto help locate gold-mining areas where the person works	
		Strenghts and weaknesses of this information system and how to re-use it	
4	Mosnier E, Garancher L, Galindo M, Djossou F, Moriceau O, Mutricy L, et al. Paludisme en Guyane: des projets de recherche opérationnelle originaux s’appuyant sur la santé communautaire. Lett Infect. 2020;35:50–78	*Article in French*	[32]
		Brief description of the Malakit strategy	
		Description of the conception of the IEC tools in a community-based approach	
5	Galindo MS, Lambert Y, Mutricy L, Garancher L, Miller JB, Gomes JH, et al. Implementation of a novel malaria management strategy based on self-testing and self-treatment in remote areas in the Amazon (Malakit): confronting a-priori assumptions with reality. BMC Public Health. 2022;22:770	Description of the logic model of the Malakit intervention	[5]
		Presentation of the process and implementation outcomes following the Conceptual Framework of Implementation Fidelity	
		Description of the factors influencing reach, participation and participants responsiveness	
6	Parent AA, Galindo MS, Lambert Y, Douine M. Combatting malaria disease among gold miners: a qualitative research within the Malakit project. Health Promot Int. 2022 Aug 1;37(4):daac058	External qualitative survey describing how Malakit is part of the trajectory of gold miners and their perception of the intervention through indivual and goup interviews and on-site observation	[9]
7	Longchamps C, Galindo MS, Lambert Y, Sanna A, Mutricy L, Garancher L, et al. Impact of Malakit intervention on perceptions, knowledge, attitudes, and practices related to malaria among workers in clandestine gold mines in French Guiana: results of multicentric cross-sectional surveys over time. Malar J. 2022 Dec 28;21:397	Evolution of knowledge, attitudes and practices among malaria before-after the Malakit of the targeted population project: positive impact on attitudes	[8]
		Comparison of of KAP between people participanting or not in Malakit using propension score: improvement of perception, knowledge and practice	
8	Douine M, Lambert Y, Galindo MS, Mutricy L, Sanna A, Peterka C, et al. Self-diagnosis and self-treatment of malaria in hard-to-reach and mobile populations of the Amazon: results of Malakit, an international multicentric intervention research project. Lancet Reg Health Am. 2021;4:100047	Presentation of the quasi-experimental design of the Malakit interventional research project, the objectives and the main indicators	[6]
		Presentation of the indicator analyses method	
		Description of the results: kit distribution, kit utilisation, impact on practices, safety data and impact on malaria epidemiology (using Interrupted Time Series)	
9	Douine M, Lambert Y, Galindo MS, Mutricy L, Sanna A, Peterka C, et al. Auto-diagnostic et auto-traitement du paludisme dans les populations isolées et mobiles de l’Amazonie: résultats de Malakit, un projet international multicentrique de recherche interventionnelle. Self-diagnosis and self-treatment of malaria in hard-to-reach and mobile populations of the Amazon: results of Malakit, an international multicentric intervention research project. Bull Epidémiologique Hebd BEH. 2022;(15):258–70	*Article in French*	[10]
		Similar to article N°8 for the French-speaking community	
		Presentation of the quasi-experimental design of the Malakit intervention research project, objectives and main indicators	
		Presentation of the indicator analysis method	
		Description of results: kit distribution, kit use, impact on practices, safety data and impact on malaria epidemiology (using interrupted time series)	
10	Lambert Y, Métras R, Sanna A, Galindo M, Hiwat H, Marchesini P, et al. Modeling the impact of Malakit intervention: one more step towards malaria elimination in the Guiana Shield? medRxiv; 2023. p. 2023.07.11.23292527. Available from: https://www.medrxiv.org/content/10.1101/2023.07.11.23292527v1	Evaluation of the impact of the Malakit strategy on malaria incidence (by species) using a modeling approach (deterministic Susceptible-Infectious-Susceptible (SIS) compartmental model)	[7]
		Estimation of number of cases averted, number of infections treated with Malakit and evolution of reproduction number	
11	Carboni C, Jimeno Maroto I, Galindo M, Plessis L, Lambert Y, Bardon T, et al. Training-of-trainers program for community health workers involved in an innovative and community-based intervention among goldminers in the Guiana Shield: a quality and effectiveness evaluation. medRxiv; 2023	Presentation of the theoretical framework conceptualized for the evaluation of training programs for the control of infectious diseases in cross-border contexts	[33]
		Details on the elaboration and realisation of the training of the CHWs of the Curema^a^ project (initial and an on-the-field training)	
		Evaluation methods for the quality and the effectiveness of the trainings	
12	Jimeno Maroto I, Galindo MS, Lambert Y, Bordalo Miller J, Carboni C, Plessis L, et al. Community engagement in mobile and hard-to-reach populations: a community-based intervention for malaria elimination in a tri-national region of the Guiana Shield. *In progress*. 2024	Presentation of the importance of integrating the community's perspectives, needs, and aspirations into planning, execution, and evaluation of the project through a community-based model	
		Description of theoretical framework of the continuum of community engagement ranging from minimal or symbolic involvement to substantive and meaningful collaboration (consulting; involving; collaborating; participating; and community led)	[20]
		Description of the elaboration IEC tools in this participatory approach	
13	Douine M, Cairo H, Galindo MS, Vreden S, Lambert Y, Adenis A, et al. From an interventional study to a national scale-up: lessons learned from the Malakit strategy at the French Guiana–Suriname border. Malar J. 2023;22:237	Description of the Malakit scale-up in the Surinamese malaria elimination programme after the the research project, with the lessons learned from this experience	[18]
14	Website www.malakit-project.org	Description of the differents components of the Malakit intervention (distribution site, step-by-step inclusion procedure, stakeholders…)	
		IEC tools availability (videos, drawings, phone app)	
		Presentation of the project to the general public through documentary films	
		Links to project publications	

^a^Curema is an ongoing project in French Guiana aimed at eliminating *P. vivax* by combining kit distribution (Malakit) with screening of potential hypnozoite carriers and their treatment with 8-aminoquinolines after G6PD assay

This article outlines the contexts in which the Malakit strategy could be transferred, describes a step-by-step approach from situation analysis, adaptation to the context and evaluation modalities to implement a tailored Malakit-like strategy in other settings.

### Defining the context in which the Malakit strategy may be of interest

The Malakit strategy is an innovative tool that cannot replace standard case management strategies performed at health care facilities or by CHWs in some settings [12]. It has been developed to provide early access to malaria diagnosis and treatment for populations that are not reached by health care and prevention services in the places where they are living and working. Malakit should be intended as the last possible option for specific populations, after careful evaluation, when all other recommended interventions have proved unfeasible. These populations may be far from the healthcare system facilities and services, working in illegal activities far from any stable human settlement for example, or moving between two countries, one of which suffers from the lack of specific intervention by its neighbor and ends up with imported cases, or when an institution cannot send healthcare workers to places where it cannot guarantee their safety.

**Fig. 2:**
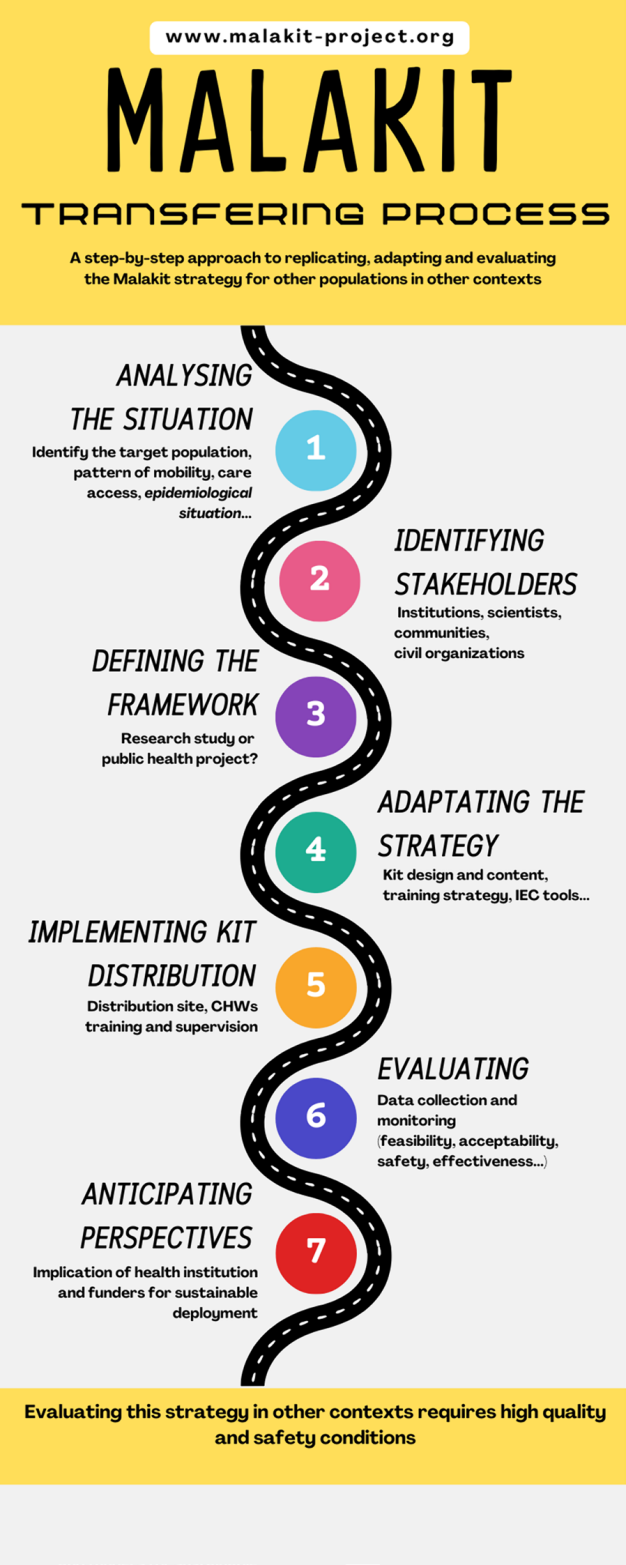
Malakit transferring process

The first step in assessing the relevance of the Malakit strategy is to characterize the epidemiological situation of malaria in the area of interest (Fig. 2). This will allow for identification of populations heavily affected by malaria that are not reached by the healthcare system. Table 2 presents the main points that should be considered [13].

**Table 2:** Information to be collected to identify areas and populations for whom the Malakit strategy would be useful

Questions to be addressed	Important information to collect
What are the characteristics of the population?	Population size
	Age structure, gender, activity, origin (cultural and linguistic norms)
	Level of education and health literacy
	Geographical situation (remoteness, accessibility, transborder context)
	Occupation, administrative status, displaced or refugee populations
	Pattern of mobility (place of work, place of living, periodicity, routes, strategic points)
	Social organization and support (community leaders, interconnections between localities and different communities)
	Identification of situations of vulnerability and social inequality
	Identification of health concerns and priorities
	Overall context: political and financial context of the country, war and civil-war, places under control of criminal organizations
What is the epidemiology of malaria in this population?	Incidence, prevalence
	Plasmodium species (proportion of *P. falciparum* and other species)
	Population most affected or at risk
	Foci and hotspots (spatial distribution)
	Seasonality and outbreaks (temporal distribution)
What are their knowledge, attitudes and practices regarding malaria?	Risk perception of malaria
	Representations and care paradigms
	Attitude in the case of malaria symptoms: use of health facilities, self-medication, traditional medicine
Why is this population not reached by the health system?	Absence of a nearby healthcare system
	Barriers to accessing the healthcare system (unsafe travel, remoteness, cost of travel)
	Cost of care
Who are the stakeholders?	Health Authorities, National/Regional Malaria Ccontrol/Elimination Programmes
	Scientists
	Health Services (public/private sector)
	Community organizations, NGO, local leaders
	Other potential actors depending on the context: social, economic, military

This assessment can be initiated using data from the surveillance system, a literature review (quantitative and qualitative studies conducted by epidemiologists, social scientists, biologists), grey literature, local knowledge from field actors and target community itself, or even mass-media information. When available data are insufficient, a cross-sectional survey and ethnographic methods evaluating these points can be considered [14].

This diagnostic stage is essential to carefully choose the optimal intervention strategy. Other interventions have been described elsewhere for case management among hard-to-reach populations, such as training CHWs [15], and have been proven as effective and safe. The strengths and weaknesses of these different strategies need to be assessed to determine whether Malakit adds any value in comparison with the sole strengthening of the health care system, or specific strategies. If *Plasmodium vivax* is predominant in a specific context, Malakit could reduce disease burden and transmission, but not prevent relapses. Malakit could thus be combined these facilities with other interventions providing radical cures such as 8-aminoquinoleines administration after G6PD deficiency screening, among potential hypnozoites carriers (based on epidemiological criteria or on *P. vivax* serology [16]). Such a strategy is currently being evaluated in the Guiana Shield (Curema project [17]).

### Specifying the operating framework

#### A need for a multiple stakeholder approach involving scientists, health institutions and the community

Based on the Malakit experience, it is recommended that stakeholders include: (i) health institutions (National malaria programs/Ministry of Health (MoH)) to support or endorse the project and specify the data that will be useful to them in deciding on sustainability/scaling-up [18]; (ii) scientists in public health, epidemiology, data science, social science and biology to carry out or support the design development, collection of good-quality data and analyses of the results [19]; (iii) representatives of the target population, such as association, community leaders or volunteers from non-organized civilian groups, and CHWs to tailor the project to the needs of the community, adapting/co-creating IEC tools (Information, Education, Communication) and empowering the community to participate in the project (which is particularly important in the elimination phase, when malaria no longer represents a major health problem for the population); (iv) health care professionals, facilities and organizations working in the area where the targeted population lives or moves, including local actors of the malaria programme who carry out standard control actions (microscopists, reporting agents) and (v) depending of the context, other stakeholders from other sectors could be involved.

Community engagement is essential. It refers to a participatory process involving interaction between entities (scientists and health institutions) and a community to integrate community perspectives, needs and aspirations into the planning, execution and evaluation of an intervention. Community engagement can be conceptualized as a continuum of participation, ranging from informing, consulting, involving, collaborating, participating, and, finally, community leading [20].

These different stakeholders may come from a single country if the target population is native to the country where they live, or from several countries if they are cross-border and/or migrant populations. The fact that this strategy is new and little tested means that partners must be strongly committed to ensuring the quality of the project and the safety of participants, thus avoiding the risk of counter-effects. A flowchart detailing the various players, their roles and their responsibilities (sponsors, investigators, coordinators, scientific committee) is very useful for formalizing the involvement of each party and can be reinforced by a contract if required.

#### Defining the shape of the implementation

If the Malakit intervention is considered to be the most appropriate response to the specific challenges posed by the fight against malaria in the region and population analysed, it can nevertheless be implemented in different ways, depending on the stakeholders involved. It could be conceived as a research project led by scientific stakeholders, as a pilot intervention conducted by a health authority or a civil society organization, or a full-scale intervention integrated within the country's health system.

A pilot project implemented in the form of intervention research has a number of advantages in the short/medium term: dedicated funding, mobilization of specific human and logistical resources and possibility of increasing the involvement of local partners (through incentives for extra workload for example). Participatory research to build alliances among institutions and communities and together can help tailoring the project [21, 22]. However, this positive aspect must be balanced with the delays needed to obtain funding and ethical approval for research activities, and by the fact that a pilot project with no prospect of being maintained or scaled up in a subsequent stage is of lower interest.

The implementation of the intervention as a pilot or full-scale intervention by field actors in charge of malaria case management within the health system could require important efforts to secure funding, administrative clearance, recruitment and/or training of relevant professionals for field operations, and it thus implies significant political commitment. But this type of implementation could ensure sustainability and a feasibility assessment within “real-life” conditions. If associated with an external, scientific evaluation with a pragmatic design, this could also fuel the scientific debate about Malakit intervention effectiveness.

#### Planning the evaluation of the intervention

Considering the principle of evidence-based public health, evaluating the intervention should be integrated as part of the Malakit strategy, either designed as a public health project or an intervention research project. Different aspects of the intervention may be evaluated, each one requiring suitable methods, outcomes and resources:

- its effectiveness on malaria epidemiology (e.g., through modelling [7, 23, 24])
- its safety, by ensuring the correct use of kits by users, or looking for unintended consequences [6, 25, 26]
- the relevance and feasibility of its extension over time and space, through implementation outcomes (acceptability, adoption, appropriateness, feasibility, fidelity, implementation cost, penetration and sustainability) [5, 27]
- its contextual framework: indeed innovation uptake depends largely on contextual factors, not just on innovation effectiveness [28].

In the context of Malakit in FG, a pragmatic approach has been developed allowing to triangulate evidence from several good quality sources of data, in order to achieve a good convergence of the required evidences [7]. High-quality data collection and monitoring systems can be implemented even in isolated and offline contexts [19]. When feasible, independent external evaluation is valuable, as long as implementation stakeholders are involved in the evaluation.

To evaluate effectiveness, experimental designs such as cluster randomized trials (and their variations) may be applicable in regions presenting a sufficient number of areas suitable for a Malakit intervention and with low inter-cluster mobility. The question of the feasibility and ethics of dividing the target population into an intervention group and a control group needs to be raised. As an alternative, stepped-wedge design, or even pragmatic or quasi-experimental designs relying on a temporal comparison for example (pre-post or time series designs) can be used. Mixed methods are highly recommended to evaluate the implementation and process and the acceptability by the stakeholders [11, 28–30].

#### Resources to be deployed

Human resource costs are probably the most important budget line to estimate when planning the implementation, supervision and coordination of a Malakit-type intervention. Different professional profiles are essential for successful implementation, including: CHWs, supervisors, project coordinators, epidemiologists/public health doctors, data managers, administrative and financial managers or social scientists. Depending on the context, the budget could include additional incentives for field workers to compensate for working in difficult and isolated contexts, or to cover the extra workload for those already salaried.

The cost of a kit in the Malakit project in the Guiana Shield was 8.40 USD, but could vary depending on local prices for pouches and supplies in other contexts. Other expenses need to be considered such as materials for facilities (small equipment) or data collection (e.g. digital tablets), service provision for IEC tools creation, travel expenses or electricity supply.

Depending on the design of the pilot phase (research or public health program), various funding sources may be considered: domestic financing, international institutions (e.g., Global Fund), private funding (e.g., foundations) or specific calls for proposals. The mobilization of human resources, particularly CHWs, is crucial to the success of this intervention. This requires a strong commitment from all the players involved, as the CHWs will be living and working in remote areas that are by essence difficult to access.

### Adapting the strategy to the context

#### Adapting the kit

The kit design needs to be adapted to the epidemiological context and to the target population sociocultural characteristics, values and norms. For example, Malakit was originally designed for adult migrants originated from Brazil, a poorly educated population living in the Amazon rainforest, so explanatory drawings were annotated in Portuguese (Fig. 1).

##### Rapid tests

For the Malakit project on the Guiana Shield, the Carestart Pan LDH RDT was used, as it was available in individual packaging, WHO and EU certified, and easy to perform and read. In 2023, this RDT was no longer available so others individually packed and WHO-prequalified tests suitable for the local epidemiology were used, even if they present a 3 or 4 bands design. The most important criterium to be considered is to use an RDT capable of diagnosing the *Plasmodium* species circulating in the given region (taking into account HRP2/HRP3 deletion), in an individual packaging, ideally with a retractable lancet (to avoid blood exposure accidents)—that can be added separately if it cannot be added in the individual packaging—temperature-stable, and with authorization for use in the region/country concerned.

##### Treatments

The choice of the treatment in the kit is based on malaria epidemiology and national protocols. The simplest is to use artemisinin-based combination therapy (ACT), which are effective on both *Plasmodium falciparum* and *P. vivax* and are recommended by the WHO, as first-line treatment for malaria attacks.

##### Age groups

In populations where malaria affects children, a "family kit" could be created, with a dosage for children based on their weight. In the case of artemether/lumefantrine (the ACT used in this project), the dose of four tablets twice a day could be easy split for children from 1 to 4 pills (4 pills being the adult dose). A table of correspondence with age and, therefore, weight (being careful in areas with malnutrition) could be proposed. When *P. falciparum* is predominant, a single low dose of primaquine for reducing transmission can be added to the treatment, with a specific dosage for children, and a warning for pregnant women [31].

##### Climate

The kit material must be adapted to the climate and the conditions of transport/use. For example, in the Amazonian context, in order to withstand humidity and transport by boat, the pouch was waterproof. Pouches for RDTs and treatments should be made of plastic to ensure the best possible storage conditions (dry and clean). The pouches containing all these items can be made locally, in waxed canvas for example. Its size should be adapted to facilitate transport, but it can be made slightly larger to hold other precious belongings such as medicines or identity papers. The more useful the kit, the more people will take care of it.

#### Adapting the intervention modalities

##### Where?

A map of the region, showing high transmission areas, the mobility of the population, transit zones and itineraries is very useful for pinpointing distribution sites. As the strategy is particularly suited to hard-to-reach populations, distribution cannot generally be carried out directly in malaria transmission areas. It is, therefore, a good strategy to set up distribution sites on routes travelled by the target population, in places where they can feel safe and take the time to receive the training (which lasts between 45 min and 1 h, depending on the person's prior knowledge of malaria). Depending on the context, it may be possible to organize the distribution of the kits directly in the target population’s home or workplace.

##### By whom?

CHWs may be responsible for distributing the kit. These must be people the population can trust, who belong to these communities or know them closely, speak the same language, be familiar with information and technology tools tools (incl. tablets, smartphones) if needed and live in or agree to move to one of the project's distribution sites. When feasible, a nursing diploma or equivalent could be an advantage. According to the experience of the Malakit project, working in pairs for CHWs enables peer learning, improves the quality of the intervention and maintains the CHWs' motivation. Depending on the context, it may also be possible to consider distributing kits in existing health facilities, if these are trusted, safe and easily accessible by the mobile target community. The CHWs are responsible for recruiting (outreach activity) and training participants, distributing kits and LLINs, replenishing or replacing kits, and collecting data. In the Malakit project, they were also responsible for assembling kits and managing stocks. Like in any complex intervention involving CHWs, this will require high-quality, effective and appropriate initial and ongoing training and supervision (see below).

##### When?

Kit distribution can be an ongoing programme throughout the year, delivering the kit to everyone who passes by. If transmission occurs at certain times of the year and mobility patterns allow it, kit distribution can be implemented on a massive scale over a shorter period before the transmission season. It could also be a solution when it is too difficult to secure human resources in these remote areas all year round. If the target population includes children or women of childbearing age, kit distribution could be combined with other programmes aimed at reducing the incidence of malaria, such as seasonal malaria chemoprophylaxis or prenatal consultations.

#### Adapting the training strategy in a community participatory approach

Training and IEC tools need to be developed at different levels: (i) at community level (target population): to inform about the project, who can participate, how and why, (ii) at the CHWs level: to train them to fully understand their role and to become trainers themselves to instruct people how to use the kits; (iii) at individual level: strategy for CHWs to train people that enhance dialogue and a verification of participant understanding. Numerous tools could be useful, based on drawings, videos or games. The development of these tools can benefit greatly from a participatory approach to adapt their usability for the community's malaria education needs [32]. This will enable the choice of communication channels (social networks, posters, radio), methods (videos, drawings, testimonials from people from the community for identification purposes) and communication codes (representations, colours, stereotypes). A smartphone application, usable off-line, has been developed in the Malakit project, containing: information on malaria and means of prevention; a video explaining how to perform an RDT; drawings and video explaining how to take the treatment; and an interactive step-by-step module to guide gold miners through the use of the kit in case of symptoms, with warning about signs of severe malaria and treatment contraindications, and regular notifications to remind when to take the kit treatment. It was not possible to assess the significance of this application regarding the correct use of the kit. Similar tools could be developed or adapted when relevant. This experience in the Malakit project taught that before launching such tools on a large scale, sufficient time should be allowed to prototype, develop and test them to ensure that their design, interface and useability actually correspond to the needs and digital literacy of the target population.

### Implementation of the strategy

#### Training of the CHWs

High-quality, effective, and appropriate training are required for effective and sustainable intervention involving CHWs [33]. This involves a great deal of effort in development, implementation, and evaluation of the training. Developing a high-quality training programme demands careful consideration of the learning methods and modules to be designed. The information contained in the CHWs Malakit training courses, the training methods and the training evaluation have been published in various articles (Table 1). The training is a good predictor of the long-term sustainability of public health initiatives and several studies have found the positive association between training and maintaining good standards of practices [34–38]. Furthermore, access to training and supervision seems to be associated with non-monetary incentive sustaining CHWs motivation and engagement [38, 39]. The initial theoretical programme is the starting point of a continuous professional development scheme, with regular re-training being as important as initial training [40]. Ongoing training, including various moments of evaluation and reflection, enable interventions to be adjusted in time to ensure proper implementation. Constant efforts throughout the project are essential to maintain quality while adapting to the inevitable changes in the context in which the intervention evolves.

#### Equipment and visibility of distribution points

The distribution points must be facilities where Malakit products can be stored in good conditions (notably temperature—usually recommended less than 35 °C—and humidity), with enough space for two to four people to be trained at the same time, and access to water for basic hygiene. Electricity for at least part of the day to charge tablets/smartphones is required, and, if an electronic data collection is planned during distribution activity, an internet connection for timely remote supervision and regular transmission of questionnaire data. These facilities must be clearly identified by the target population as distribution sites, for example with banners or posters mentioning the distribution periods of time, and easily accessible.

#### Distribution procedure

After explaining the aim and purpose of the project, CHWs train the person, ideally one by one but sometimes with up to four persons at the same time. The aim is for the person receiving the kit to know the symptoms of malaria, how to perform an RDT, how to take the treatment and the symptoms of severity requiring urgent care. Self-performance of the RDT is one of the crucial points. If the test is positive, the participant can be referred to the nearest malaria care service or treated by the CHWs according to his abilities and authorizations. Interactivity, multimedia tools and teach back are very useful to support the training. When a participant comes back to a distribution site after having used the kit, the CHWs can refill or replace the kit. According to the strategy framework, CHWs can be responsible for data collection at both first visit or return visits. Supplemental Materials 1, and previous publications [4, 5] describe the inclusion process in details.

#### Logistics circuit

A logistics circuit must be carefully designed to ensure the availability of all kit components, and the traceability of drugs and RDTs (batch numbers and expiration dates). It is preferable to rely on a pre-existing circuit at regional or national level to facilitate ordering (e.g., via a pre-existing platform). Stock management and conservation must be monitored at the central level and at each facility. The kit can be assembled centrally or directly at distribution sites.

#### Supervision

Strong supervision is needed to: (i) ensure continuous training to CHWs; (ii) ensure that the delivery of the intervention is satisfactory, available, accessible and adequate; (iii) ensure that tests and drugs are used correctly; and (iv) exchange information to facilitate coordination between stakeholders. It is important to establish a relationship of trust with CHWs to give them support and recognition for their work and learn from their knowledge gained in the field as they are key people in this type of intervention. Indeed, ongoing training and supervision is linked to improvement of quality service and to motivation [40].

#### Risks and challenges

The risks of such an intervention must be assessed and controlled as far as possible. Incorrect use of the kit can lead to a delay in treatment, which can be detrimental for the person. If treatment is taken with poor compliance on a large scale, there is a risk of selecting resistant parasites. Medicines can be a source of adverse effects. Involving the healthcare system in the project to report misuse and adverse effects, as done in this project, may prove useful.

The ethical aspect of CHWs' behaviour ensuring that kit distribution is free of charge and paying close attention to the resale of kits by those receiving them, is important. Measures to reduce this risk, such as mass distribution to avoid a parallel market, or monitoring people who come too frequently to receive a kit, can be useful tools.

### Assessment and perspectives

Whether the strategy is implemented as a research project or as a public health intervention, data collection is necessary to determine whether it is worth continuing the strategy over time or extending it spatially. As mentioned above, various methods can be used to evaluate the feasibility, effectiveness and safety of the intervention. The links between the data collected during the intervention and the national malaria control program and/or surveillance system need to be discussed from various angles, such as the integration of the strategy into the long-term program, the integration of data from the Malakit strategy into the data surveillance system, and the assessment of the cost-effectiveness of the strategy in specific contexts. This last point is important as an argument for sustainability and financial backing. The cost of the strategy must be weighed against the reduction in morbidity (care, hospitalization, sick leave), malaria transmission (positive knock-on impact), employment creation and economic development (reduction in the economic impact of the burden of malaria, local fabrication of the Malakit pouches), capacity-building within the community (improved prevention and care practices), etc. These prospects for continuation and geographical extension must be anticipated from the outset of the project. To achieve this, decision-makers must be involved in the project from the outset (e.g. the country's health institutions or potential funders such as the Global Fund) [18]. There are a number of guidelines to drive the implementation of generalization, even if not all the consequences can always be anticipated [18, 41–43]. Table 3 highlights the key points to ensure the quality of Malakit's replicability.

**Table 3:** Key points for implementing a Malakit strategy

• Carry out a preliminary diagnosis of the situation (population, mobility, epidemiology)	
• Only consider Malakit if no other internationally recommended strategy offering access to case management is possible	
• Build a trust relationship with the target population	
• Develop information and training tools adapted to the target population	
• Include in the kit RDTs and treatments suited to the epidemiological context	
• Ensure training and close supervision of CHWs recruited to be close to the community	
• Ensure that participants are properly trained in the use of the kit	
• Ensure that the kit is free of charge for the participants	
• Provide the means allowing to evaluate participants' use of the kit, their safety, and overall strategy relevance in the specific context	

## Conclusion

The Malakit strategy is a new approach that overturns the way malaria case management is think, out of the dogma of the doctor-patient relationship which strongly involve target populations and community health workers. The Covid-19 pandemic has also accelerated the acceptance of home testing, not only by individuals themselves (self-test), but also by the health professionals realizing outreach activities [44]. The Malakit strategy help make a significant step towards the elimination of malaria. As this is a complex intervention, involving many different components, its transferability remains an open question. By adapting the intervention to the needs of a different context while respecting the main points listed in Table 3, Malakit can help other countries to pursue their elimination efforts. In the transferability process of complex interventions, a balance needs to be found between adaptation—modifying aspects that are context-specific—and the adherence to core features which represent the very heart of the intervention, and which should be maintained. In this light, this article identifies the core principles that represent the very essence of the Malakit experience to guide the reflection of the readers about aspects that need adaptation to local needs and resources.

Although malaria is an ancient disease, its epidemiology and populations at risk are constantly evolving. With climate change and increased population movements due to armed conflict, economic or environmental crises, the fight against malaria must be innovative, adaptable and ambitious [45, 46]. The evaluation of the Malakit strategy transferability and effectiveness in new contexts will be essential to increase and refine the evidence of its value, and to decide whether Malakit-like interventions could be an additional tool in the arsenal recommended in future WHO guidelines.

### Supplementary Information

Supplementary Material 1.

## Data Availability

Not applicable

## Abbreviations

CHWs: Community Health Workers
RDTs: Rapid diagnostic tests
ACT: Artemisinin-based combination therapy
ASGM: Artisanal and small-scale gold mining
IEC tools: Information, education, communication
MoH: Ministry of Health
