# Supplementary material for: Core principles of Malakit intervention for transferability in other contexts
Source: Malar J. 2024 Jun 13;23:185. doi: 10.1186/s12936-024-05002-0 (PMC11170856; doi:10.1186/s12936-024-05002-0)
Supplement: Supplementary file 1 — Supplementary Material 1. [file 12936_2024_5002_MOESM1_ESM.docx]

# Supplementary Material 1: Inclusion process of individuals by CHWs

## Steps to enroll subjects in the Malakit study

After explaining the aim and purpose of the project, CHWs train the person, ideally one-by-one but might be up to four persons at the same time. The training can be supported by illustrated posters, games, videos.

1. Check eligibility criteria for targeting people belongings to the targeted community
2. Provide information using IEC tools on the project to the potential participant(s):

- The enrollment process takes between 30 minutes and one hour
- Participation involves pricking oneself on the finger as part of the learning process facilitated by the CHW on how to use the rapid test
- Participants must sign a consent form (if relevant, for example in the framework of research project )

1. Display the kit and its components
2. Conduct short discussions about malaria with participants to evaluate their knowledge then completing the training with the objectives for the participants:

- To understand that the self-care kit is a relief assistance to be used in situations isolated from a health center and otherwise it is always better to seek professional care.
- To understand the importance of correct use of the kit, and receive additional information about malaria and how to prevent it,
- To learn how to perform a self-diagnostic test, read and interpret the results, and treat themselves accordingly and appropriately.
- To understand the contraindication and the severity symptoms needing a rapid consultation in health facilities

1. Guide the participant as they perform a step-by-step self-administered RDT, with the help of the printed illustrations on the kit and/or the video and then interpret the results. Self-administration of the entire RDT process is one of the inclusion criteria. *If the test is positive, the participant can be referred to the nearest malaria care service or treated by the CHWs according to his abilities.*
2. Explain how to take the Malakit medications according to the test result. This includes the concept of resistance and the importance of finishing the entire course of treatment, even if the person feels better. If relevant, contraindications for taking single dose primaquine, such as being pregnant or breastfeeding, are also explained, using the illustration on the kit.
3. Collect data through a standard questionnaire according to the tools used (tablet, paper…) according to the design of the Malakit implementation
4. Distribution of the phone app if applicable

## Steps for a return visit

When a participant returns to a distribution site, it is useful when feasible to collect information on how they have used the kit before refilling or replacing it. this enables the individual's training to be verified and reinforced.

1. Debrief the participant on what has happened since the last visit at a distribution site (episodes of symptoms, use of the kit including use of the kit by someone else…)
2. Replace or replenish the kit if necessary.
3. Administer the return visit questionnaire.

NB: The facilitator has the right to refuse to replenish or provide a new kit if he finds out the person is being deceitful and only wants to obtain kits to sell.
